# Kassporin-KS1: A Novel Pentadecapeptide from the Skin Secretion of *Kassina senegalensis*: Studies on the Structure-Activity Relationships of Site-Specific “Glycine-Lysine” Motif Insertions

**DOI:** 10.3390/antibiotics11020243

**Published:** 2022-02-13

**Authors:** Yueyang Lu, Wanchen Zou, Lei Wang, Xinping Xi, Chengbang Ma, Xiaoling Chen, Tianbao Chen, Chris Shaw, Xu Zhang, Mei Zhou

**Affiliations:** 1 School of Medicine & Holisitc Integrative Medicine, Nanjing University of Chinese Medicine, Nanjing 210023, China; ylu21@qub.ac.uk; 2 School of Pharmacy, Queen’s University Belfast, 97 Lisburn Road, Belfast BT9 7BL, UK; wzou02@qub.ac.uk (W.Z.); l.wang@qub.ac.uk (L.W.); x.xi@qub.ac.uk (X.X.); c.ma@qub.ac.uk (C.M.); x.chen@qub.ac.uk (X.C.); t.chen@qub.ac.uk (T.C.); chris.shaw@qub.ac.uk (C.S.)

**Keywords:** antimicrobial peptides, motif, temporin, structure-activity relationships

## Abstract

Due to the abuse of traditional antibiotics and the continuous mutation of microbial resistance genes, microbial infections have become serious problems for human health. Therefore, novel antibacterial agents are urgently required, and amphibian antimicrobial peptides (AMP) are among the most interesting potential antibacterial leads. In this research, a novel peptide, named kassporin-KS1 (generically QUB-1641), with moderate antibacterial activity against Gram-positive bacteria, was discovered in the skin secretion of the Senegal running frog, *Kassina senegalensis*. Using site-specific sequence enrichment with a motif “glycine-lysine” that frequently occurs in ranid frog temporin peptides, a series of QUB-1641 analogues were synthesized, and effects on selected bioactivities were studied. The greatest activity enhancement was obtained when the “glycine-lysine” motif was located at the eighth and ninth position as in QUB-1570.QUB-1570 had a broader antibacterial spectrum than QUB-1641, and was eight-fold more potent. Moreover, QUB-1570 inhibited *S. aureus* biofilm most effectively, and significantly enhanced the viability of insect larvae infected with *S. aureus*. When the “glycine-lysine” motif of QUB-1570 was substituted to reduce the helix ratio and positive charge, the antibacterial activities of these synthetic analogues decreased. These data revealed that the “glycine-lysine” motif at positions 8 and 9 had the greatest enhancing effect on the antibacterial properties of QUB-1570 through increasing positive charge and helix content. This research may provide strategies for the site’s selective amino acid modification of some natural peptides to achieve the desired enhancement of activity.

## 1. Introduction

Since the widespread availability of antibiotics beginning in the 1950s, antibiotics have been widely used clinically. Antibiotics can help to cure acute and refractory infectious diseases and promote the implementation of clinical treatments, such as chemotherapy and organ transplantation [1]. However, due to the uneven quality of antibiotics, the abuse of antibiotics, and the lack of supervision of drug use, the resistance of microorganisms to antibiotics arose in clinical cases simultaneously. The continuous emergence of super-bacteria and the difficulty in curing related infectious diseases indicates that drug-resistant bacterial infections have become a major hazard to human health [2,3]. Thus, it has become an urgent imperative to develop innovative antibacterial drugs.

Antimicrobial peptides (AMPs) are derived from immune defense substances in most vertebrates [4]. Most positively charged AMPs destroy the microbial membrane structures by combining with the negatively charged phospholipid layer on these membranes [5]. Conventional antibiotics generally kill microorganisms by specifically inhibiting the production of certain proteins [6]. For example, penicillin inhibits peptidoglycan synthesis by regulating TacL to disintegrate microbial cell walls [7]. Therefore, AMPs can kill microorganisms more directly than common antibiotics. Moreover, it is relatively complex for microorganisms to develop resistance to AMPs since the process to change their membrane composition is highly complicated [8]. Consequently, AMPs with such properties have become promising antibacterial lead compounds for drug development.

Amphibians are the main source of natural AMPs [9]. *Kassina senegalensis*, a kind of terrestrial frog, is widely distributed in sub-Saharan Africa [10]. Many biologically active peptides from this frog exhibit pharmacological activity on microorganisms, cancer cell lines, inflammatory mediators, and smooth muscles of isolated rat organs [11,12,13,14,15]. Therefore, researchers have speculated that *Kassina senegalensis* contains potential biologically active peptides worth exploring through in-depth study.

Novel amphibian AMPs have been continually discovered over several decades [16]. Most AMPs have been classified into peptide “families” based on their features of primary structures and common properties of other peptides, but the peptides from *Kassina senegalensis* are different. When these peptides were searched in peptide databases such as BLAST (https://blast.ncbi.nlm.nih.gov/Blast.cgi, accessed on 25 October 2021), there were no peptides that shared highly conserved sequences with them. Hence, the peptides from *Kassina senegalensis* possess unique sequences, and have not been classified into certain families. From the analysis of sequences and antibacterial results, some peptides in *Kassina senegalensis* show similarities to some temporin family peptides.

Temporins constitute one of the largest AMP families, and nearly 150 members have been described as of the end of 2020. Among the smallest AMPs in Nature, temporin was first obtained from the *Rana temporaria* frog’s skin in 1996 [17]. They have relatively short amino acid sequences (less than 14 residues), carry fewer positive charges at neutral pH value, and contain alpha-helix secondary structures. As a result of post-translational modification, the C-terminals of temporins are amidated [18].

Through bioinformatics analyses of the members of the temporin peptide family, it was discovered that they have a high proportion of hydrophobic amino acids [19]. From another point of view, “Phenylalanine-leucine-proline-” (FLP-) usually appears at the N-terminus of temporins [20]. The 3D-structural predictions of temporins imply that the phenylalanine and leucine residues at positions 1 and 2 promote the extension of the hydrophobic face, and the proline residue at position 3 helps form α-helix [21]. Moreover, phenylalanine or leucine residues frequently occur at the C-terminals of temporins, and positively charged amino acids are often located centrally in the peptides [19].

Most temporins specifically inhibit Gram-positive bacteria by destroying their membranes at low concentrations (less than 20 µM) [22]. This is mainly due to the difference in membrane structure between Gram-negative bacteria and Gram-positive bacteria [20]. The membrane structure of Gram-negative bacteria consists of an inner membrane and an outer membrane. The lipopolysaccharide on the outer membrane contains negatively charged phosphate groups and divalent cations, and their neutralizing effects interfere with the electrostatic adsorption of temporins to phospholipid membranes [23]. Compared with Gram-negative bacteria, Gram-positive bacteria with negatively charged plasma membranes can more easily attract positively charged temporins as the first step in their killing mechanism [24].

This study reports the isolation, structural and functional characterization of a novel AMP, named kassporin-KS1 (QUB-1641), from the skin secretion of the African hyperoliid frog, *Kassina senegalensis*. This temporin-like peptide has exhibited moderately potent inhibition of Gram-positive bacteria, and has the potential to be optimized by structural engineering into a highly effective analogue. The “glycine-lysine” motif from temporin peptides was incorporated at different sites into QUB-1641 to synthesize a group of such structurally modified analogues. The peptides were tested for their antibacterial efficacy, anti-biofilm effects, hemolysis effects, permeabilization of microbial membranes, and the systemic treatment of bacterially infected larvae compared to the synthetic replicate of the natural peptide.

## 2. Results

### 2.1. “Shotgun” Cloning of the Kassporin-KS-1 (QUB-1641) Precursor Encoding cDNA from a cDNA Library of Kassina Senegalensis Skin Secretion

**Figure 1:**
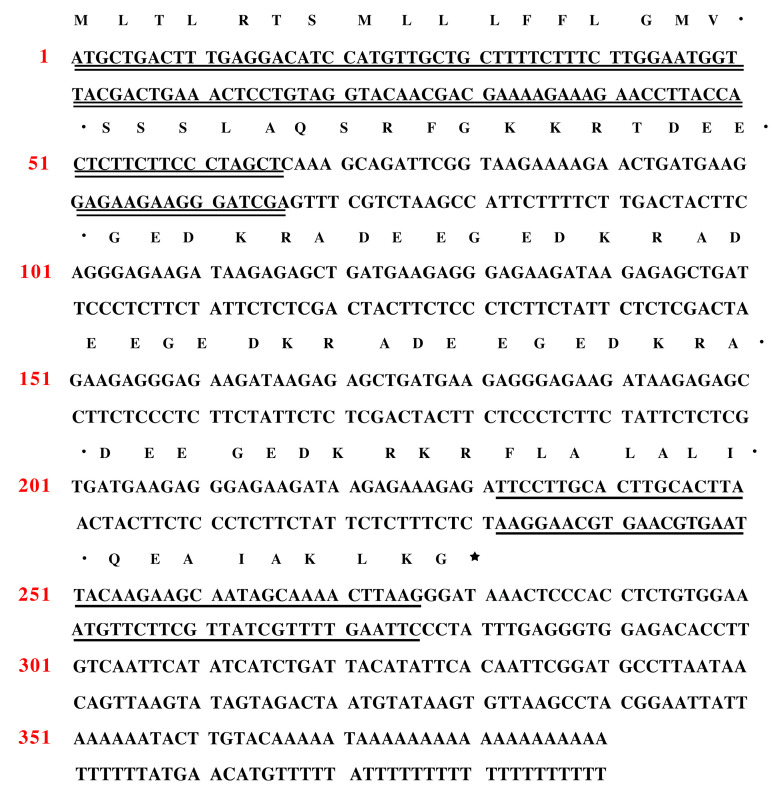
Nucleotide and translated amino acid sequences from the open reading frame of the biosynthetic precursor of novel peptide, kassporin-KS1, from a *Kassina senegalensis* skin secretion derived from the cDNA library. Double underlining, single underlining, and an asterisk are used to indicate the signal peptide, mature kassporin-KS1 (QUB-1641), and stop codon, respectively.

The full-length cDNA encoding a novel peptide kassporin-KS1 (QUB-1641) was obtained from the *Kassina senegalensis* skin secretion cDNA library by molecular cloning. The novel peptide-encoding precursor nucleotide sequence and the corresponding translated amino acid sequence are presented in Figure 1. The entire open reading frame consisted of a signal peptide of 22 highly conserved amino acids, an acidic spacer region, and a mature peptide encoding domain of 15 amino acids. A putative protein convertase with lysine-arginine cleavage selectivity produced the N-terminus of the mature peptide. The mature peptide’s C-terminal lysine residue was likewise amidated via a specific protease using glycine as the amide donor. The nucleotide sequence of the kassporin-KS1 (QUB-1641) precursor was archived in Genbank (Accession number: OM243852).

### 2.2. The Identification and Sequence Characterization of Kassporin-KS1 (QUB-1641)

**Figure 2:**
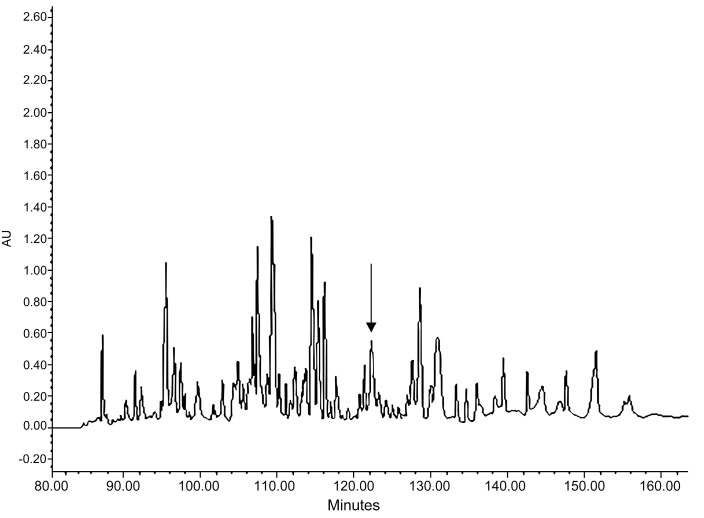
RP-HPLC chromatogram of *Kassina senegalensis* skin secretion at 214 nm. The fraction containing novel peptide, kassporin-KS1 (QUB-1641), is indicated by an arrow. The retention time was 122 min.

**Figure 3:**
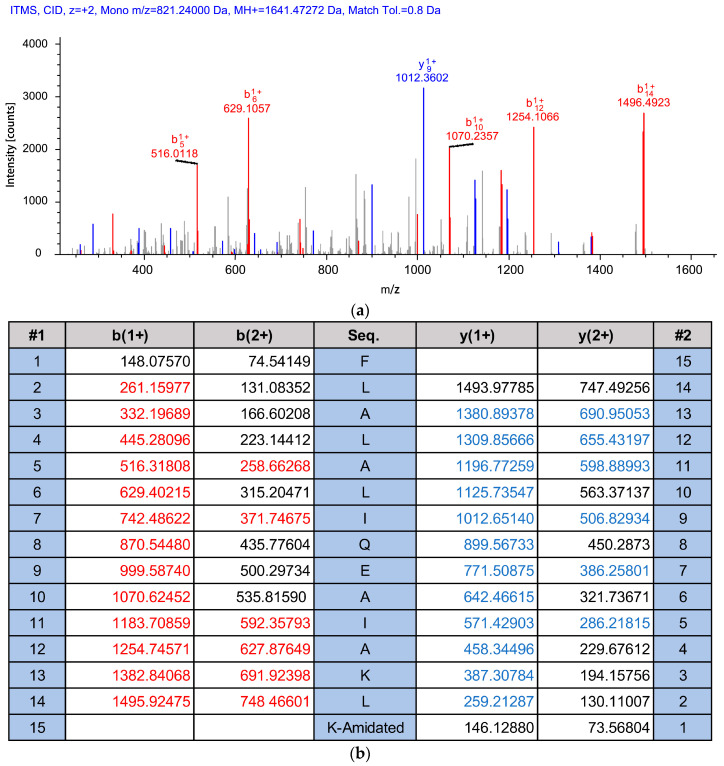
The MS/MS fragment mass spectrum of doubly charged ions of kassporin-KS1 (QUB-1641) (**a**) and the MS/MS ion dataset predicted from the biosynthetic precursor (**b**). The predicted b-ions and y-ions are respectively marked in red and blue. The “Seq.” column in the table was the amino acid sequence of the novel peptide.

The *Kassina senegalensis* skin secretion was fractionated by RP-HPLC (Figure 2). As indicated by the arrow, the retention time of kassporin-KS1 was 122 min. The MS/MS fragment mass spectrum showed that the molecular mass of the peptide contained in #122 was 1641.472 (Figure 3a). Kassporin-KS1 was thus given the systematic designation based on confirmed molecular mass as QUB-1641. The sequence of this peptide was established by MS/MS fragmentation sequencing. (Figure 3b). The amino acid sequence of the peptide with C-terminal amidation was consistent with the observed molecular mass and the molecular cloning data.

### 2.3. Design of Kassporin-KS1 (QUB-1641) Analogues

**Figure 4:**
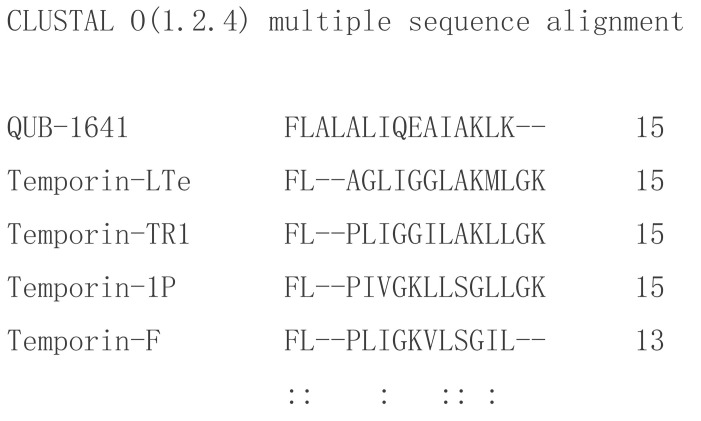
The sequences of kassporin-KS1 (QUB-1641) and some partially similar temporins were compared using Clustal Omega. The colons indicate similar residues.

**Figure 5:**
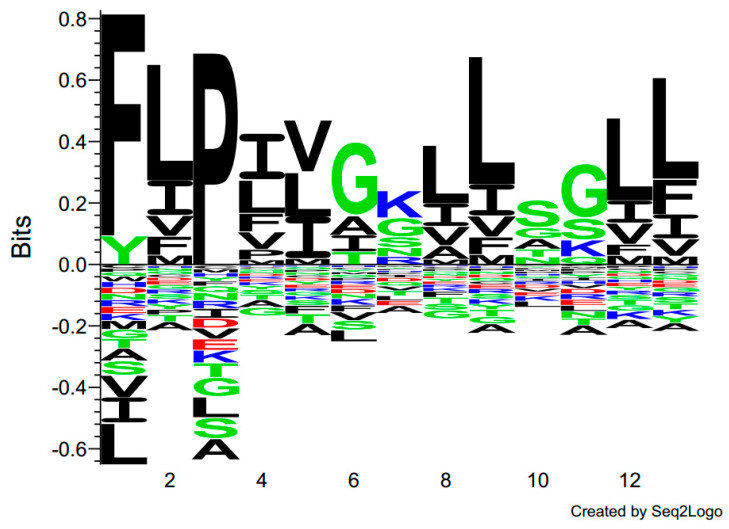
The amino acid sequences of 120 temporins were recorded in Seq2logo. The amino acid diversity at each position is sorted in descending order.

The sequence of kassporin-KS1 (QUB-1641) was searched using the Basic Local Alignment Search Tool (BLAST), and the results indicated that the novel sequence was not identical to any archived entry. However, through the use of the multiple sequence alignment program Clustal Omega, kassporin-KS1 (QUB-1641) was found to have some similarities to a few temporin peptides (Figure 4). They were all short peptides with Phenylalanine-leucine- at their N-terminals, and some shared leucine, isoleucine, lysine residues within their sequences. As a result, the original systematic name, QUB-1641, was given an alternative name, kassporin-KS1. The inhibitory activities of these temporins on Gram-positive bacteria were significantly higher than those of QUB-1641 [25]. According to the analysis of the primary structures of many temporins, using the Seq2Logo sequence logo generator, glycine and lysine residues appeared in the sixth and seventh positions of temporins with high frequency (Figure 5). It has also been reported that the changes in glycine and lysine can regulate the activity of other AMPs [19].

According to the findings above, the structure of kassporin-KS1 (QUB-1641) was modified at different positions by inserting the “glycine-lysine” motif of the temporins. The effect of the modification on a range of bioactivities was studied using designed analogues, QUB-1599, QUB-1585, QUB-1570, and QUB-1643 (Table 1). QUB-1570, synthesized by replacing the residues at the eighth and ninth positions of QUB-1641 with “glycine-lysine”, produced the best antibacterial analogue. To study the changes in the physical and chemical properties of the “glycine-lysine” motif at respective positions, the amino acids in the motif were changed. The “glycine-lysine” motif of QUB-1570 was changed to “glycine glycine” and “proline lysine” in analogues QUB-1498 and QUB-1609, which both led to a decrease in the cationicity of the peptides and destruction of the helix. These two QUB-1570 analogues participated in all the related assays to compare induced changes.

### 2.4. The Purification and Identification of Kassporin-KS1 (QUB-1641) and Analogues

The chromatograms of the synthesized peptides purified by RP-HPLC are shown in Figure S1. The MALDI-TOF MS spectra verified the purity of the peptides (Figure S2). The mass-to-charge ratios of the labelled peptide-containing peaks were identical to the expected molecular masses of the peptides.

### 2.5. Physicochemical Properties and Structural Analysis of QUB-1641 and Its Analogues

**Figure 6:**
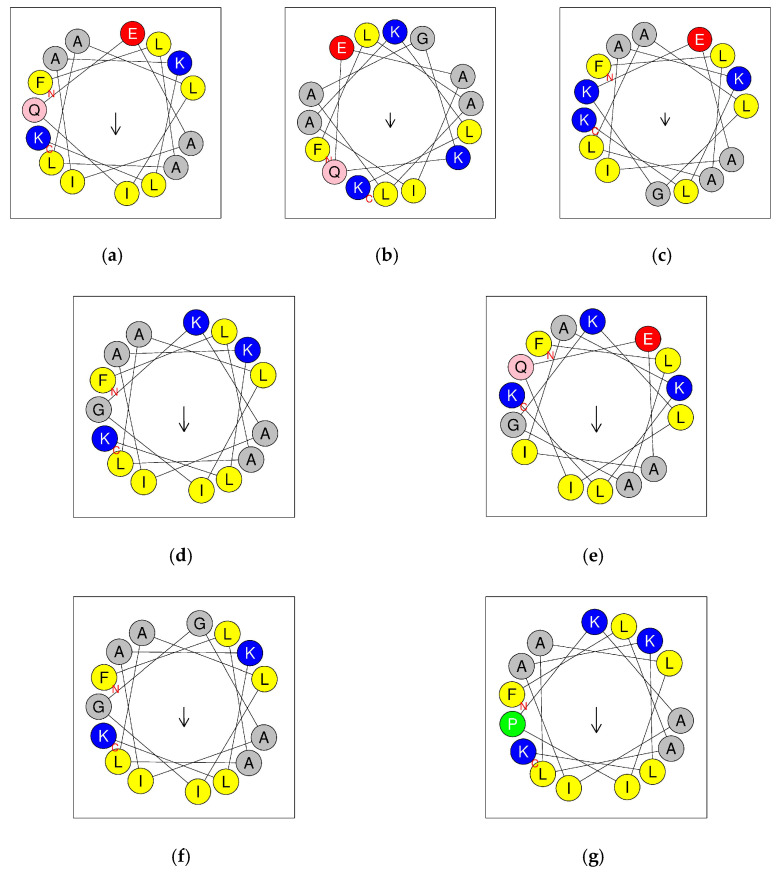
Helical wheel diagrams of kassporin-KS1 (QUB-1641) and synthetic analogues: QUB-1641 (**a**), QUB-1599 (**b**), QUB-1585 (**c**), QUB-1570 (**d**), QUB-1643 (**e**), QUB-1498 (**f**), and QUB-1609 (**g**). Positively charged residues are marked in blue, hydrophobic residues are yellow, negatively charged residues are red, aromatic residues are green, uncharged polar residues are pink, other non-polar residues and glycine are marked in gray. The arrow represents the direction of the hydrophobic moment.

**Figure 7:**
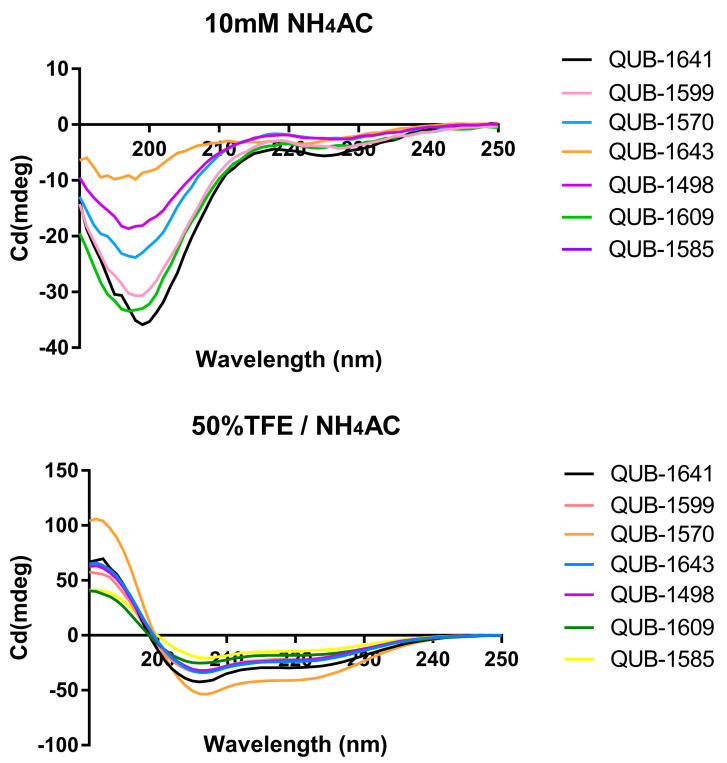
QUB-1641 and its analogues were dissolved at 50 µM in 10 mM NH_4_Ac solution (pH 7.4) and in 50% TFE/10 mM NH_4_Ac solution (pH 7.4) for the CD spectra.

The amino acid distributions and hydrophobic moments of QUB-1641 and its analogues are shown in Figure 6. The CD spectra of these peptides are presented in Figure 7. Table 2 summarizes the properties of the peptides. Compared with QUB-1641, the positive charges of QUB-1641 analogues increased, while their hydrophobicity and helix percentages increased or decreased, respectively. QUB-1570 had the most cationic and highest helix ratio among the peptides. The CD spectra of tested peptides in the 10 mM NH_4_Ac solution presented a broad negative peak at 207 nm, indicating that the peptides all exhibited random coil structures in the aqueous environment. In 50% TFE/10 mM NH_4_Ac solution, all peptides showed a positive peak at 192 nm and negative peaks at 208 and 222 nm, indicating that the peptides exhibited α-helical structures in membrane-mimicking environments.

### 2.6. Mean Inhibitory Concentration (MIC)/Mean Bactericidal Concentration (MBC) Assays of Kassporin-KS1 (QUB-1641) and Its Analogues

The antibacterial activities of kassporin-KS1 (QUB-1641) and its analogues against representative microbes are shown in Table 3. QUB-1599 was the only analogue with no inhibitory effect on all the microbes. Other analogues showed improved antibacterial activities, and their antibacterial spectra also broadened. The MICs and MBCs of QUB-1570 and QUB-1609 against *S. aureus* were eightfold more potent than QUB-1641. The modification of QUB-1570 was the most successful, and this peptide specifically eliminated Gram-positive bacteria at 8 µM.

### 2.7. Anti-Biofilm Activity of Kassporin-KS1 (QUB-1641) and Its Analogues

The effects of kassporin-KS1 (QUB-1641) and its analogues on the biofilms of *Staphylococcus aureus* (*S. aureus*) and *Escherichia coli* (*E. coli*) are shown in Table 4. QUB-1641 had moderate antibacterial effects, reflecting a weaker effect on the microbial biofilms. Compared with QUB-1641, the anti-biofilm activities of the analogues were improved. With the exception of QUB-1599, the analogues specifically inhibited *S. aureus* biofilm growth. The MBICs of the analogues against *S. aureus* and *E. coli* were significantly higher than their MICs. QUB-1498 and QUB-1609 inhibited the *S. aureus* biofilm at 32 μM, but the mature *S. aureus* biofilm was only eliminated when the concentration reached 128 μM. Among all the peptides, QUB-1570 was the most effective in eliminating stubborn biofilms. QUB-1570 destroyed the *S. aureus* biofilm at 16 µM. Moreover, almost all peptides lost their activities on the biofilms of *E. coli*, but QUB-1570 was able to clear *E. coli* biofilm at 64 μM.

### 2.8. Killing Kinetics of Kassporin-KS1 (QUB-1641) and Its Analogues

**Figure 8:**
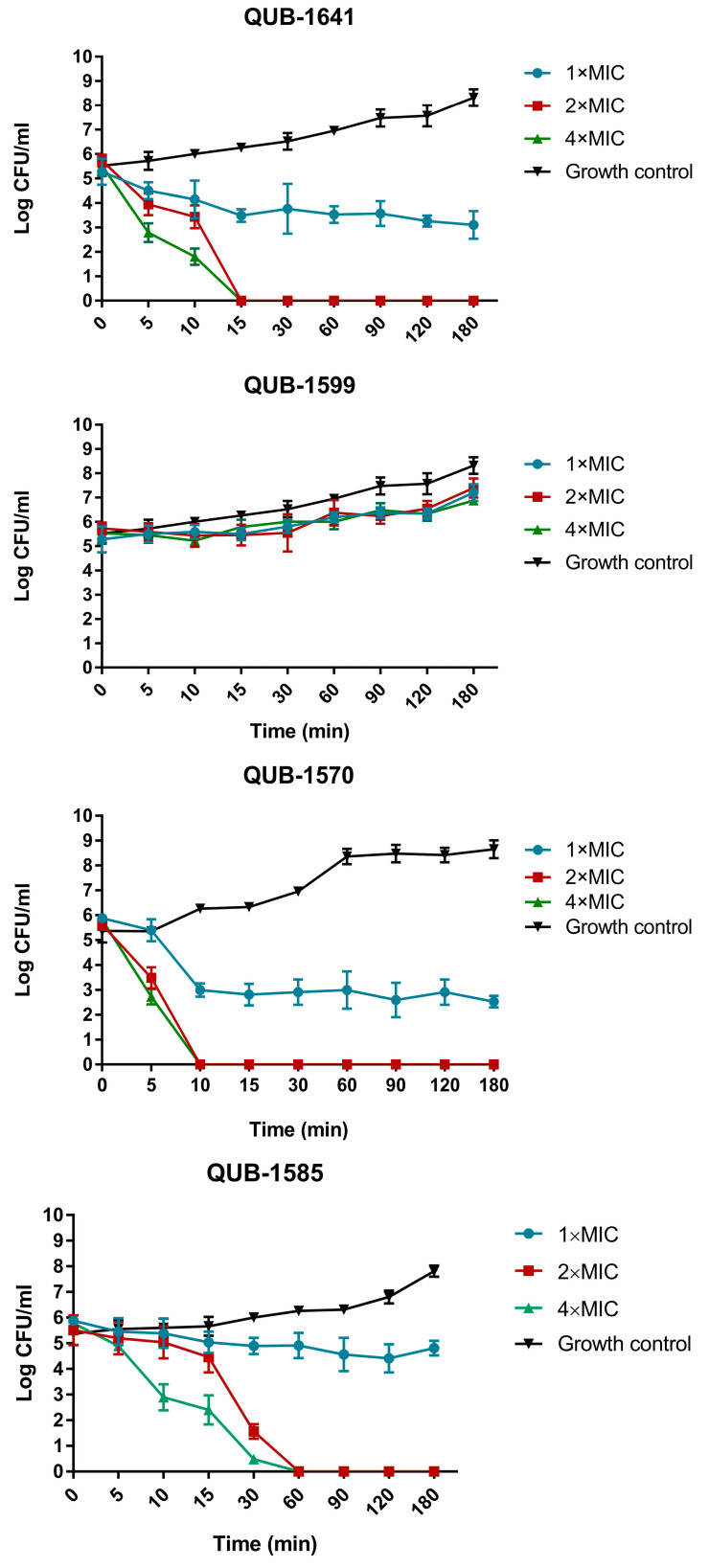

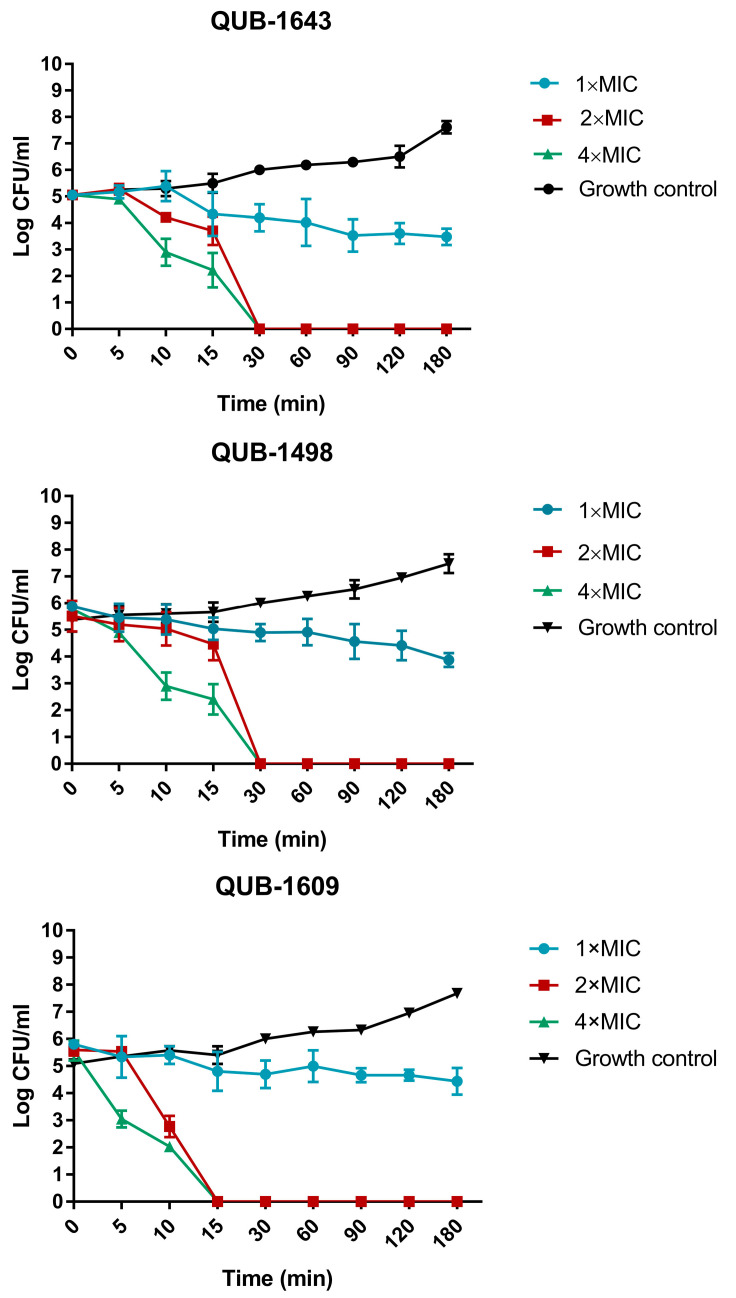
Time-kill line charts of kassporin-KS1 (QUB-1641) and its analogues against *S. aureus*. The logarithmic values of colony-forming units (CFU) at 0, 5, 10, 15, 30, 60, 90, 120, and 180 min were reflected on the y-axis. The growth control was the bacterial-medium suspension at the same time point. Error bars represent the standard error of the average number of microbial cells measured in CFU/mL at each time point (based on five replicates).

QUB-1641 and its analogues (except QUB-1599) inhibited the growth of *S. aureus*. Thus, their bactericidal efficiency against *S. aureus* was tested (Figure 8). Besides QUB-1599, all peptides eliminated *S. aureus* within 60 min. The antibacterial effect of QUB-1641 was relatively weak, but it quickly eliminated *S. aureus* within 15 min at 2 × MIC. When the concentration of QUB-1641 was 1 × MIC, its inhibitory effect on *S. aureus* was relatively slow, and it did not achieve the complete killing effect. QUB-1585 was the slowest effective peptide to eliminate *S. aureus*, and its elimination rate was not affected by concentration. QUB-1643, QUB-1498, and QUB-1609 cleared *S. aureus* within 30 min at 2 × MIC. Among all the 2 × MIC peptides, QUB-1570 had the fastest inhibitory rate against *S. aureus*, achieving microbial clearance within 10 min.

### 2.9. Membrane Permeability of QUB-1641 and Its Analogues

**Figure 9:**
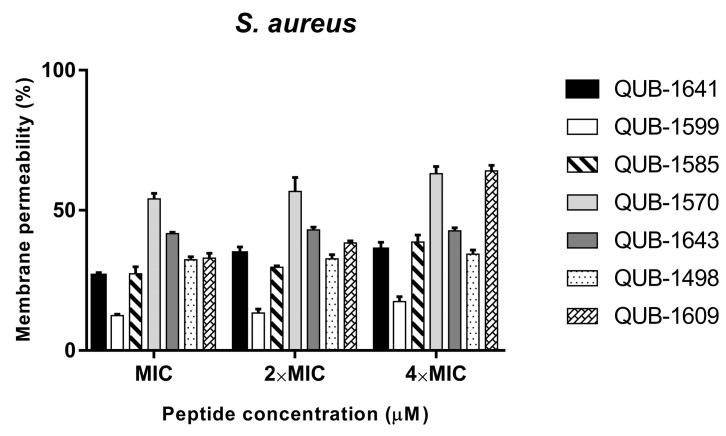
Membrane permeability of QUB-1641 and its analogues against *S. aureus*. The positive control was 70% isopropyl alcohol and bacterial suspension. The data represented the ± SEM of five replicates.

To study the antibacterial mechanism of the peptides, the permeability of QUB-1641 and analogues to the *S. aureus* membrane was investigated (Figure 9). The membrane permeability of QUB-1599 was less than 20%, which is significantly weaker than other peptides. The membrane permeation ratio of QUB-1585 increased with the concentration, but did not exceed 50% at the highest concentration. The peptide concentration did not affect the membrane permeability of QUB-1641, QUB-1643, and QUB-1498. QUB-1570 and QUB-1609 had the strongest permeability to the *S. aureus* membrane. QUB-1570 caused membrane permeability of more than 50% at the MIC, and as the concentration increased to 4 × MIC, its membrane permeability was only 10% higher than the corresponding value of the MIC. For QUB-1609, the increase in membrane permeability was proportional to its peptide concentration.

### 2.10. Salt and pH Sensitivity of Kassporin-KS1 (QUB-1641) and Its Analogues

The antibacterial activities of QUB-1641 and its analogues against *S. aureus* under different pH values and ion environments are shown in Table 5. The MICs and MBCs of most peptides were significantly increased in these environments. In the weak acid or weak base environment, the peptides modified with the “glycine-lysine” motif almost lost their antibacterial activities, while the MICs of QUB-1498 and QUB-1609 were not greatly influenced. Besides QUB-1570, motif-modified peptides lost their antibacterial activities in salt environments. The antibacterial activity of QUB-1570, QUB-1498, and QUB-1609 at the physiological salt concentration was approximately two to four times weaker than that of the status without salts. Surprisingly, the antibacterial activity of QUB-1498 and QUB-1609 in the MgCl_2_ solution increased.

### 2.11. Hemolytic Activity of Kassporin-KS1 (QUB-1641) and Its Analogues

**Figure 10:**
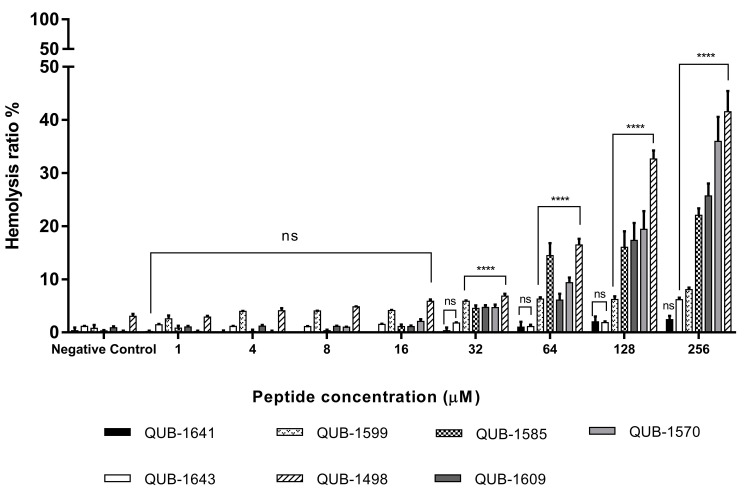
The hemolytic activities of kassporin-KS1 (QUB-1641) and its analogues. Triton X-100 was the positive control. PBS solution was the negative control. The results of the peptide sample group and the negative control group were compared by using two-way ANOVA. The significance level was: **** = *p* < 0.0001, ns = no significant difference. The error bars indicated the ± SEM of five replicates.

The hemolytic activity of all tested peptides was less than 20% when the peptide concentration was at or below 64 µM (Figure 10). When the peptide concentration was higher than 64 μM, the hemolytic activity of QUB-1570 and QUB-1498 increased significantly, and was nearly 40% at 256 μM.

### 2.12. The Influence of Peptides on the Survival Rate of Wax Worm Larvae Infected with S. aureus

**Figure 11:**
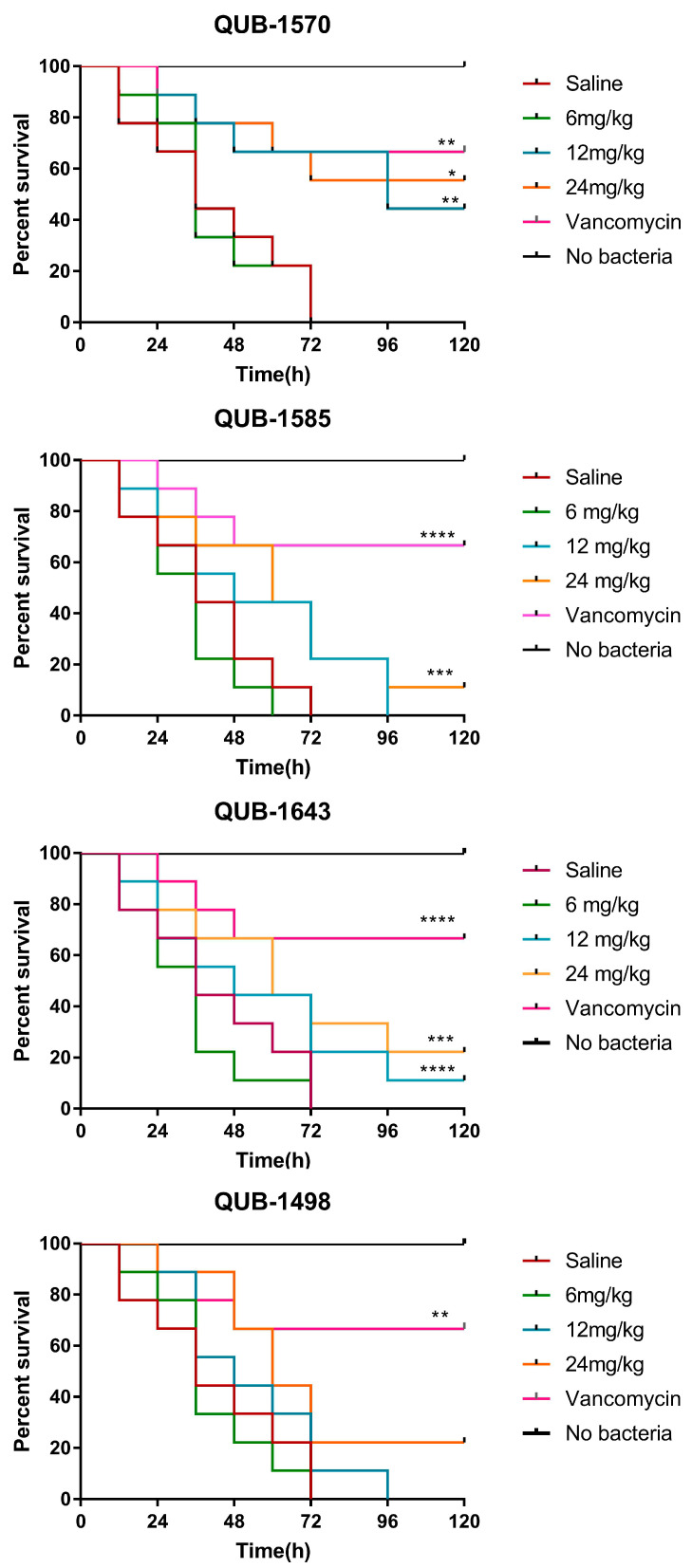

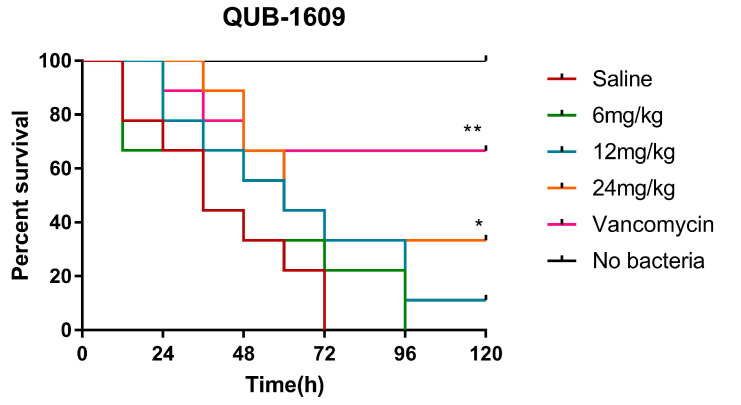
Treatment of *S. aureus* infected *Galleria mellonella* larvae by kassporin-KS1 (QUB-1641) analogues. Infected larvae were treated with peptides at 6, 12, and 24 mg/kg, respectively. The negative control was the saline injection to the larvae, and the positive control was the administration of 20 mg/kg Vancomycin. ‘*’ indicated Log-rank (Mantel–Cox) Test of each peptide sample group against the negative control: * *p* < 0.05, ** *p* < 0.01, *** *p* < 0.001, **** *p* < 0.0001.

Different concentrations of peptides (6, 12, 24 mg/kg) were dosed after the larvae were infected with *S. aureus*; their survival ratio was then evaluated (Figure 11). The peptide concentrations were determined based on the MBC of the peptides against *S. aureus*. QUB-1641 was difficult to dissolve in saline, causing the larvae to die directly after injection. QUB-1599 had no effect on *S. aureus*. Therefore, these two peptides were not included in this assay. After saline injection, the larvae infected with *S. aureus* survived no more than 72 h. Among the tested peptides, only QUB-1609 prolonged the survival time of larvae at 6 mg/kg. At 12 mg/kg, QUB-1585 and QUB-1498 resulted in 0% larval survival at the end of 96 h, whereas QUB-1643 and QUB-1609 both promoted the larvae to survive over 120 h, yet with a low survival rate of less than 20%. However, QUB-1570 sustained the survival of over 40% of the larvae for 120 h at 12 mg/kg while causing a survival rate close to that of the positive control at 24 mg/kg, achieving the best therapeutic effect among all of the homologues.

## 3. Discussion

Amphibian antimicrobial peptides (AMPs) have become the lead compounds expected to replace conventional antibiotics due to their low resistance generation and abundant sources [26]. However, natural amphibian AMPs still have defects in their pharmacological activity, cytotoxicity, and in vivo stability. To bring AMPs closer to clinical applications, structural modifications aimed at optimizing the pharmacological activities of natural AMPs have become the focus of many researchers.

In this study, a novel biologically active peptide, kassporin-KS1 (QUB-1641), was identified in the skin secretion of *Kassina senegalensis*. Although the peptide was unique in sequence, it shared similarities with some temporins, such as displaying the “glycine-lysine” motif commonly found in temporins. Therefore, the core content of our research was to use this motif to optimize QUB-1641 and study structure-activity relationships. The motif “glycine-lysine” has been found to have positive effects on the antibacterial activity of AMPs.

From the composition of amino acids in the motif, the long side-chain of lysine helps improve the permeability of peptides in microbial membranes [27]. In addition, the positive charge carried by lysine promotes the tight binding of AMPs with the negatively charged phospholipid layer through electrostatic adsorption [28].

For the glycine contained in the motif, due to its simple structure, its conformation has excellent stability. The presence of a single glycine could reduce the accumulation of conventional helical structures, but it can also interfere with a helical structure’s stability [29,30]. Therefore, substituting “glycine-lysine” at different positions of kassporin-KS1 will cause the diversity of peptide analogue structures, and their antibacterial activities should also be different. It has been reported that when glycine is located in the middle of short peptides, it can better reduce steric hindrance, improve the conformational freedom, and enhance the cell selectivity of the peptides [31]. This may be the reason QUB-1570 achieved the best antibacterial effect.

After that, the analogues QUB-1498 and QUB-1609 were synthesized based on the sequence of QUB-1570. QUB-1498 had replaced the lysine at position 9 with glycine. Therefore, QUB-1498 had one less positive charge, and its helical content was also significantly reduced. For QUB-1609, the cationic was retained, but the glycine at position 8 was replaced with proline. The helical structure of QUB-1609 was disrupted due to the presence of proline [32]. In the subsequent antibacterial activity assay, the activities of QUB-1498 and QUB-1609 were both weaker than QUB-1570. This indicated that the “glycine-lysine” motif increased the charges and helix content by replacing the QUB-1641’s eighth and ninth amino acids to enhance antibacterial activity.

Amino acid compositions, number of positive charges, and hydrophobicity affect the activity of AMPs [33,34]. In the MIC/MBC assays, although QUB-1599 contained two positive charges and a helical structure, its inhibitory effect on microorganisms was negligible. It was noteworthy that, compared with other analogues, the hydrophobicity of QUB-1599 was the weakest. This revealed that positive charges are not the only factor determining the antibacterial activity. The peptides with the most prominent enhancement of antibacterial effect were QUB-1570 and QUB-1609. The changes in their physical and chemical parameters indicated that the balance between positive charges, helical content, and hydrophobicity could reduce the MICs and MBCs of AMPs.

It is not appropriate to judge these peptides’ antibacterial effect as drugs based on the MIC and MBC values of the peptides in the planktonic state. The effect of antibacterial agents is usually reduced after microorganisms form biofilms [35]. The biofilms affect the pH value, oxygen content and ion concentration of the microenvironment to reduce antibacterial agents’ effect and cause drug resistance [36]. In the anti-biofilm assay, the selected strains, *Staphylococcus aureus* and *Escherichia coli* are common ESKAPE pathogens that constitute biofilms [37]. Once these microorganisms attach to venous catheters, breathing tubes, prostheses, and other implants, patients will suffer from infections, inflammation, and delayed wound healing [38]. For QUB-1498 and QUB-1609, there is a significant difference between their MBIC and MBEC values. This may be because the initial formation of biofilms is reversible and relatively easily inhibited. However, mature biofilms are relatively stubborn, and their elimination can only be achieved under the action of higher peptide concentrations [39]. QUB-1570 exhibited the strongest anti-biofilm activity among all the tested peptides. Therefore, QUB-1570 has the potential to become a lead compound against microbial biofilms.

In the membrane permeability assay, all peptides permeabilized *S. aureus* at their MICs, indicating that the antibacterial mechanisms of QUB-1641 and modified peptides are related to the destruction of microbial membrane structure. The penetration ratio of QUB-1570 to *S. aureus* was higher than other analogues. Thus, QUB-1570 was speculated to cause microbial cell death by affecting microbial membranes, similar to most temporins [25].

After exploring the peptides’ antibacterial mechanisms on *S. aureus*, the time-killing kinetics of the peptides on *S. aureus* were also tested. Most peptides at the MICs did not completely kill *S. aureus* since the bacteria’s continuous growth rate exceeded the inhibitory efficiency of peptides on the microorganisms during the assay. All peptides were able to eliminate *S. aureus* at 2 × MICs. This was probably since the 2 × MICs of the peptides against *S. aureus* were close to their MBCs, achieving more effective killing. Among these, 2 × MIC of QUB-1570 eliminated *S. aureus* within 10 min. Combined with the membrane permeability assay results, it could be concluded that QUB-1570 achieves antibacterial effects by rapidly destroying microbial membranes.

Based on the discoveries above, the anti-biofilm activities of QUB-1570 might be attributed to its rapid elimination of bacteria, which could destroy the initially formed biofilm, prevent further accumulation of colonies, and accelerate the lysis of mature biofilms. QUB-1570 could be applied in targeted therapy of diseases related to *S. aureus* infection.

However, the antimicrobial activity sensitivity of peptides at different physiological concentrations of ions restricted their development as potential clinical drugs. With the exceptions of QUB-1498 and QUB-1609, the peptides synthesized with the “glycine-lysine” motif showed poor antimicrobial activity in acidic, alkaline, or ionic environments. Their antibacterial activity was reduced by at least fourfold. This may be caused by the high sensitivity of lysine to pH changes [40].

Interestingly, the physiological concentration of magnesium ions weakly increased the inhibition of *S. aureus* by QUB-1498 and QUB-1609, which did not have the “glycine-lysine” motif. Magnesium ions have been reported to reduce the formation of biofilms and act synergistically with AMPs to promote greater curvature on their membranes. It affects the membrane structures of microorganisms, and ultimately leads to membrane leakage [41]. Nonetheless, low concentrations of magnesium ions could also induce the attachment of microorganisms [42]. This implied that magnesium ions have selective effects on AMPs, which explains why magnesium ions can enhance the antibacterial activity of QUB-1498 and QUB-1609 but not QUB-1570.

In addition to assessing peptide antibacterial activities, peptide effects on erythrocytes as surrogate body cells should also be considered to measure possible cytolytic effects. The analogues’ antibacterial activity had improved, and most of them did not cause significant hemolysis within the MBC range for Gram-positive bacteria. QUB-1570 and QUB-1498 caused a higher proportion of hemolysis above 64 μM. This may be due to their helical content and hydrophobicity interfering with normal red blood cell membranes at high concentrations [43].

In the in vivo assay, the larvae possessed innate immune systems similar to mammals, therefore they were the preferred infection model [44]. QUB-1570 increased the survival ratio of *S. aureus* infected larvae to more than 50% at 24 mg/kg, close to the survival rate induced by the positive control. This showed that the most successfully modified peptide, QUB-1570, achieved decent efficacy in the initial treatment of animal models infected with *S. aureus*.

Overall, through the modification of the novel AMP, kassporin-KS1, by the “glycine-lysine” motif, the motif’s influence on structure-activity relationships was clarified, and opened up new ideas for the optimal design of short active peptides. On the one hand, the analogue QUB-1570, synthesized by replacing the eighth and ninth amino acid of kassporin-KS1 with “glycine-lysine”, had the best potential to treat *S. aureus*-related infections. This peptide will be further modified to be closer to the standard of clinical application. On the other hand, it is necessary to further prove the effectiveness of the “glycine-lysine” motif by expanding its application scope, such as optimizing other short peptides.

## 4. Materials and Methods

### 4.1. Acquisition of Amphibian Skin Secretions

*Kassina senegalensis* frogs were kept for four months before harvesting their skin secretions (*n* = 3, male and female mixed). The back of the frogs was gently massaged and transdermal electrically stimulated. Then, the skin secretions were flushed with deionized water into the beaker. After that, skin secretions were quickly lyophilized into powder by liquid nitrogen and stored in a −20 °C freezer. This experiment complied with the UK Animal (Scientific Procedures) Act 1986’s relevant regulations, and was reviewed by the Animal Care and Use Institutional Committee of Queen’s University Belfast on 1 March 2011.

### 4.2. “Shotgun” Cloning Kassporin-KS1 (QUB-1641) Biosynthetic Precursor Encoding cDNA from the cDNA Library Established from Kassina Senegalensis Skin Secretions

The polyadenylated mRNA of the lyophilized skin secretion of *Kassina senegalensis* was isolated. The isolated mRNA was reverse transcribed into cDNA using Dynabeads^®^ mRNA Direct^TM^ kit (Dynal Biotech, Merseyside, UK), and the corresponding cDNA library was set up by rapid amplification PCR (RACE-PCR). The complete peptide nucleotide sequence was achieved through the SMART-RACE cDNA amplification kit (Clontech, Palo Alto, CA, USA). Nested universal primers (NUP) and sense primers (AC-S AGC AGC AAA AGA AGA AGA AGC CAT G) were used in 3′-RACE reactions to obtain the target sequence. The purification and cloning of RACE products were then provided by the application of the pGEM^®^-T Easy Vector System (Promega, UK). The nucleotide sequence of the cloned product was analyzed by the ABI PRISM^®^ 3100 Genetic Analyzer (Applied Biosystems, Foster City, CA, USA).

### 4.3. Isolation of Kassporin-KS1 (QUB-1641) from the Skin Secretion of Kassina Senegalensis

Approximately 5 mg *Kassina Senegalensis* skin secretion was dissolved in 0.5 mL buffer (water/ trifluoroacetic acid (TFA), 99.95/0.5, *v*/*v*) and centrifuged. The supernatant was then pumped into the Reversed Phase High Performance Liquid Chromatography (RP-HPLC) with Jupiter^®^ C18 Column (250 × 10 mm, Phenomenex, Macclesfield, UK). Skin secretions were eluted by a linear gradient program from water/TFA (99.95/0.05, *v*/*v*) to water/TFA/acetonitrile (19.95/0.05/80, *v*/*v*/*v*). The RP-HPLC ran at 1 mL/min for 240 min and automatically collected fractions every minute. The supernatant of the centrifuged skin secretions was also pumped into the LCQ Fleet Ion Trap Mass Spectrometer. Peptide masses and sequences were obtained through their mass-to-charge ratio and the dissociation fragments. Proteome Discoverer 1.0 software was applied to identify the mass and amino acid sequence of QUB-1641.

### 4.4. Chemical Synthesis of Kassporin-KS1 (QUB-1641) and Its Analogues

Sufficient peptides involved in the research were synthesized by Fmoc Solid-phase peptide synthesis (SPPS) [45]. Each amino acid in the peptide chain was mixed with hexafluorophosphate benzotriazole tetramethyl urea (HBTU) and Rink Amide resin for synthesizing catalysis and the amidation of the peptide’s C-terminus. Afterwards, the synthesis reaction undergoes dimethylformamide (DMF) washing, piperidine deprotection, N-methyl morpholine (NMM) dissolution and activation in the peptide synthesizer. Finally, the entire peptide chain completed the connection from the C-terminus to the N-terminus. The remaining resin and peptide sidechain-protecting groups were then cleaved. The reagents required for cleavage contain 2% thioanisole (TIS), 2% ddH_2_O, 2% 1,2-ethanedithiol (EDT), and 94% TFA. After the impurities were filtered, the peptide was precipitated, washed, and lyophilized. Then, the purified peptide and its analogues were collected by RP-HPLC, and the purity of the peptides was determined by Matrix-Assisted Laser Desorption/Ionization Time-of-Flight Mass Spectrometry (MALDI-TOF MS).

### 4.5. Structural Investigation of Kassporin-KS1 (QUB-1641) and Analogues

The amino acids and sequences of kassporin-KS1 (QUB-1641) and its analogues were analyzed by the online tool Heliquest (http://heliquest.ipmc.cnrs.fr, accessed on 25 October 2021). The peptides’ secondary structures were predicted by circular dichroism (CD) spectroscopy. The peptide samples were dissolved in 10 mM ammonium acetate (NH4Ac) and trifluoroethanol (TFE)/10 mM NH4Ac (50/50, *v*/*v*) to form 50 μM solutions. Then, the quartz cuvette containing the peptide solution was placed in the JASCO J-815 spectropolarimeter (JASCO Inc., Easton, MD, USA) at 20 °C for structural analysis at 190–250 nm (bandwidth: 1 nm, instrument scanning speed: 100 nm/min, data interval: 0.5 nm). Finally, the sample value was obtained through three-pass scanning accumulation. Then, the helix ratio of these peptides was tested on BestSel (https://bestsel.elte.hu/index.php, accessed on 25 October 2021).

### 4.6. The Minimum Inhibitory Concentration (MIC) and Minimum Bactericidal Concentration (MBC) Tests of Kassporin-KS1 (QUB-1641) and Analogues

The inhibition of representative microorganisms by peptides was evaluated by the modified broth dilution technique. The microorganisms included *Candida albicans* (*C. albicans*, NCTC 10231), *Enterococcus faecalis* (*E. faecalis*, NCTC 12697), *Staphylococcus aureus* (*S. aureus*, NCTC 10788), *Klebsiella pneumoniae* (*K. pneumoniae*, ATCC 43816), *Methicillin-resistant Staphylococcus aureus* (*MRSA*, ATCC 12493), *Pseudomonas aeruginosa* (*P. aeruginosa*, ATCC 27853), and *Escherichia coli* (*E. coli*, NCTC 10418).

Firstly, the selected microorganism was incubated in conical flasks with 100 mL Mueller Hinton broth (MHB) overnight. Then, the bacterial solution was sub-cultured in 20 mL MHB until it reached the logarithmic growth phase. Meanwhile, the peptide solution was prepared. The peptide was dissolved into 128 × 10^2^ μM solution with phosphate-buffered saline (PBS) and then double-diluted into 64, 32, 16, 8, 4, 2, and 1 × 10^2^ μM. Each sample solution and positive control (2 g/mL Norfloxacin) was added to 96-well plates and divided into five replicates, 1 μL per well. The blank control was 100 µL MHB, and the growth control was 100 µL bacterial solutions. When the optical density (OD) value corresponding to the microbial concentration reached the value (Gram-positive bacteria: 0.23; Gram-negative bacteria: 0.4; fungi: 0.15) in their logarithmic growth phase, the microorganisms were diluted to the required concentration with MHB (Gram-negative and Gram-positive bacteria: 5 × 10^5^ CFU/mL; fungi: 1 × 10^6^ CFU/mL).

The diluted bacterial solution was then added to the 96-well plate at 99 µL per well. The plate was incubated at 37 °C and observed the next day. The MIC was the corresponding lowest peptide concentration without microbial growth. The absorbance of the 96-well plate at 550 nm was measured by Synergy HT Microplate Spectrophotometer (Biotech, Minneapolis, MN, USA), and the obtained values were quantitatively analyzed by Prism 7 software. Subsequently, the concentrations corresponding to the clear solution were removed from the 96-well plate in 10 µL and dropped onto the solidified MHA. The Petri dishes with MHA were cultured overnight, observed and the minimum peptide concentration without colonies was MBC.

### 4.7. Anti-Biofilm Assays

As previously described, the MBIC and MBEC of the peptides against bacteria were determined [46]. *Staphylococcus aureus* (*S. aureus*, NCTC 10788) and *Escherichia coli* (*E. coli*, NCTC 10418) was selected as representative strains. For the MBIC assay, the cultures were sub-cultured to their logarithmic growth phases. The diluted bacterial suspension was incubated with the peptide solution overnight in a 96-well plate. The resulting biofilm was washed with PBS solution, fixed with methanol, and dried. The biofilm was then stained with 0.1% (*w*/*v*) crystal violet, washed again with PBS, and dissolved in 30% acetic acid. The absorbance of the solution in each well at 595 nm was counted by the Synergy HT Microplate Spectrophotometer (Biotech, Minneapolis, MN, USA). Compared with the negative control, the lowest concentration that inhibits biofilm formation was MBIC. For the determination of MBEC, the diluted bacterial solution was incubated in the 96-well plate for 24 h, with 100 µL per well. Then, the biofilm was washed and re-incubated with 100 µL peptide-medium solution. After 24 h, the biofilm was again washed, stained, and dissolved as in the MBIC assay. Each well was detected at 595 nm by the same plate reader in MBIC. Compared with the negative control, the lowest concentration to eliminate biofilm was MBEC.

### 4.8. Membrane Permeability Assays

*Staphylococcus aureus* (*S. aureus*, NCTC 10788) was transferred from MHB to TSB for sub-culture until it reached its logarithmic growth phase. After centrifuging the bacterial suspension, the bacterial cells were collected. The bacteria were washed twice according to the steps of adding 5% TSB, centrifuging, and discarding the cell supernatant. Then, the washed bacteria were mixed with 5% TSB until the absorbance of the bacterial suspension at 590 nm reached the OD value of 1 × 10^8^ CFU/mL. In total, 50 µL of bacterial suspension and 40 µL peptide solution were blended in each well of a light-shielded 96-well plate for 2 h at 37 °C. The positive control was the suspension of bacteria completely lysed with 70% (*v*/*v*) isopropanol. Each well was then filled with 10 µL SYTOX green. The fluorescence intensity of the wells was tested using a microplate spectrophotometer (Biotech, Minneapolis, MN, USA) at 485 nm excitation and 528 nm emission wavelengths.

### 4.9. Killing Kinetics Assays

The cultured *Staphylococcus aureus* (*S. aureus*, NCTC 10788) was sub-cultured to the logarithmic growth phase and diluted to 5 × 10^5^ CFU/mL. The peptide solution and the bacterial suspension were mixed into MIC, 2 × MIC, and 4 × MIC solutions. Then, the solution was aspirated at 10 µL from the tube at 0, 5, 10, 15, 30, 60, 90, 120, and 180 min, diluted with PBS, and dropped onto Petri dishes containing MHA. After incubating overnight, viable counts were performed on the cultured colonies.

### 4.10. Salt and pH Sensitivity Assays

As mentioned in the previous study, the salt and pH sensitivities of AMPs exposed to physiological concentrations of salts or non-neutral environments were tested [47]. MHB containing different ion concentrations (150 mM NaCl, 1 mM MgCl_2_, and 4 μM FeCl_3_) and MHB with pH 6.0 and pH 8.0 were prepared. The selected microorganism *Staphylococcus aureus* (*S. aureus*, NCTC 10788) was incubated overnight in the prepared medium, sub-cultured to the logarithmic phase, and then diluted to 5 × 10^5^ CFU/mL. The subsequent steps were carried out as the MIC/MBC assay.

### 4.11. Hemolysis Assays

As described previously [45], two mL of red blood cells were gently removed from the lower layer of fresh horse blood and transferred to 50 mL tubes. Then, the horse erythrocytes were washed by adding PBS and centrifuging at 1000× *g* repeatedly until the supernatant turned clear. The PBS solution in the tube was finally replenished and mixed to form 4% (*v*/*v*) erythrocyte suspension. Meanwhile, peptides were diluted with PBS to form peptide solutions and then serially diluted to the lowest concentration. The negative control was PBS solution, and the positive control was 1% Triton X-100. Then, 200 µL of 4% erythrocyte suspension was added to the 1.5 mL tubes containing 200 µL peptide solution or the control and incubated at 37 °C. After two hours, the tubes were taken out and centrifuged at 900× *g*. Then, 100 µL of supernatant were removed to a new 96-well plate. Their absorbance at 550 nm was detected by the microplate spectrophotometer (Biotech, Minneapolis, MN, USA).

### 4.12. Evaluation of the Efficacy of Peptides on S. aureus in Wax Worm Models In Vivo

*Galleria mellonella* larvae of equal weight were injected with bacterial suspensions to observe the peptides’ efficacy [45]. First, the wax worm larvae were weighed (Livefood UK Ltd., Rooks Bridge, UK). The appropriate weight for the assay was 250 ± 25 mg. Then, *S. aureus* NCTC 10788 was cultured in sterilized saline until the bacterial suspension density reached 10^8^ CFU/mL. The larvae injected with the bacterial suspension were observed for viability within 2 h. Then, 10 µL peptide solutions were injected into the larvae at 6, 12, and 24 mg/kg. The positive control was the dosage of 20 mg/kg Vancomycin. The negative control was the administration of saline. In total, nine wax worms were included in each group. After being injected, the number of larvae deaths was recorded every 12 h until it reached 120 h.

### 4.13. Statistical Analysis

Graph Pad Prism 7.00 was applied to analyze all of the data (Graph Pad Prism Inc., La Jolla, CA, USA). Error bars reflected the standard error (SEM) of five iterations. The larvae models were evaluated using the log-rank test. Significant differences were indicated by * (*p* < 0.05), ** (*p* < 0.01), *** (*p* < 0.001), **** (*p* < 0.0001) and ns (no significant difference).

## Figures and Tables

**Table 1:** The sequences of QUB-1641 and its analogues.

Peptide	Sequence
QUB-1641	FLALALIQEAIAKLK-NH2
QUB-1599	FLALAGKQEAIAKLK-NH_2_
QUB-1585	FLALALGKEAIAKLK-NH_2_
QUB-1570	FLALALIGKAIAKLK-NH_2_
QUB-1643	FLAGKLIQEAIAKLK-NH_2_
QUB-1498	FLALALIGGAIAKLK-NH_2_
QUB-1609	FLALALIPKAIAKLK-NH_2_

**Table 2:** The physicochemical properties of kassporin-KS1 (QUB-1641) and its analogues. The α-helix (%) of the peptides was calculated using the online tool Bestsel (http://bestsel.elte.hu/results.php, accessed on 25 October 2021) based on CD spectra.

Peptide	Sequence	Hydrophobicity	Hydrophobic Moment	Net Charge	α-Helix (%)
QUB-1641	FLALALIQEAIAKLK-NH2	0.706	0.267	+1	45.3
QUB-1599	FLALAGKQEAIAKLK-NH_2_	0.407	0.178	+2	36.7
QUB-1585	FLALALGKEAIAKLK-NH_2_	0.535	0.154	+2	30.8
QUB-1570	FLALALIGKAIAKLK-NH_2_	0.697	0.290	+3	67.4
QUB-1643	FLAGKLIQEAIAKLK-NH_2_	0.506	0.303	+2	40.8
QUB-1498	FLALALIGGAIAKLK-NH_2_	0.763	0.224	+2	37.6
QUB-1609	FLALALIPKAIAKLK-NH_2_	0.745	0.295	+3	33.0

**Table 3:** MICs and MBCs of kassporin-KS1 (QUB-1641) and its analogues.

Microorganism	MIC/MBC (µM)						
	QUB-1641	QUB-1599	QUB-1585	QUB-1570	QUB-1643	QUB-1498	QUB-1609
*S. aureus* NCTC 10788	64/64	>128/>128	32/64	2/8	32/64	16/16	8/32
*E. coli* NCTC 10418	>128/>128	>128/>128	64/128	16/32	128/>128	32/>128	8/64
*C. albicans* NCTC 10231	>128/>128	>128/>128	64/128	8/8	64/128	32/>128	16/32
*MRSA* NCTC 12493	64/128	>128/>128	64/128	4/8	64/128	128/>128	8/16
*K. pneumoniae* ATCC 43816	>128/>128	>128/>128	>128/>128	32/32	>128/>128	>128/>128	128/>128
*E. faecalis* NCTC 12697	64/128	>128/>128	128/>128	8/8	128/>128	32/64	16/64
*P. aeruginosa* ATCC 27853	>128/>128	>128/>128	>128/>128	>128/>128	>128/>128	>128/>128	>128/>128

**Table 4:** Anti-biofilm activity of kassporin-KS1 (QUB-1641) and its analogues against *S. aureus* and *E. coli*.

Microorganism		MBIC/MBEC (µM)					
	QUB-1641	QUB-1599	QUB-1585	QUB-1570	QUB-1643	QUB-1498	QUB-1609
*S. aureus* NCTC 10788	128/>128	>128/>128	64/128	4/16	128/>128	32/128	16/128
*E. coli* NCTC 10418	>128/>128	>128/>128	128/128	32/64	>128/>128	>128/>128	64/128

**Table 5:** MICs and MBCs of kassporin-KS1 (QUB-1641) and analogues in physiological salts and different pH environments.

Medium	MIC/MBC (µM)						
	QUB-1641	QUB-1599	QUB-1585	QUB-1570	QUB-1643	QUB-1498	QUB-1609
PH 7.0	64/64	>128/>128	32/64	2/8	32/64	16/16	8/32
PH 6.0	>128/>128	>128/>128	>128/>128	128/>128	>128/>128	32/64	16/64
PH 8.0	>128/>128	>128/>128	>128/>128	64/128	128/>128	32/64	32/64
+NaCl (150 mM)	>128/>128	>128/>128	128/>128	8/16	128/128	16/64	16/64
+MgCl_2_ (1 mM)	128/>128	>128/>128	128/>128	16/32	128/>128	8/32	4/16
+FeCl_3_ (4 µM)	>128/>128	>128/>128	128/>128	16/32	128/>128	16/64	8/64

## Data Availability

The kassporin-KS1 biosynthetic precursor-encoding cDNA has been deposited in the NCBI Database under an accession number: OM243852.

## Supplementary Materials

The following supporting information can be downloaded at: https://www.mdpi.com/article/10.3390/antibiotics11020243/s1, Figure S1: The RP-HPLC chromatogram of the crude peptides, the peak indicated by the arrow contains the pure peptides (QUB-1641 (a), QUB-1599 (b), QUB-1585 (c), QUB-1570 (d), QUB-1643 (e), QUB-1498 (f), and QUB-1609 (g)); Figure S2: The purified peptides’ MALDI-TOF mass spectra (mass-to-charge ratios) (QUB-1641 (a), QUB-1599 (b), QUB-1585 (c), QUB-1570 (d), QUB-1643 (e), QUB-1498 (f), and QUB-1609 (g)) contained in the labelled peaks in Figure S1.
