# Supplementary material for: Kassporin-KS1: A Novel Pentadecapeptide from the Skin Secretion of Kassina senegalensis: Studies on the Structure-Activity Relationships of Site-Specific “Glycine-Lysine” Motif Insertions
Source: Antibiotics (Basel). 2022 Feb 13;11(2):243. doi: 10.3390/antibiotics11020243 (PMC8868508; doi:10.3390/antibiotics11020243)
Supplement: Supplementary file 1 [file antibiotics-11-00243-s001.zip › antibiotics-1584889-supplementary.pdf]

Kassporin-KS1: A Novel Pentadecapeptide from the Skin Secretion of Kassina senegalensis: Studies on the Structure-Activity Relationships of Site-Specific "Glycine-Lysine" Motif Insertions

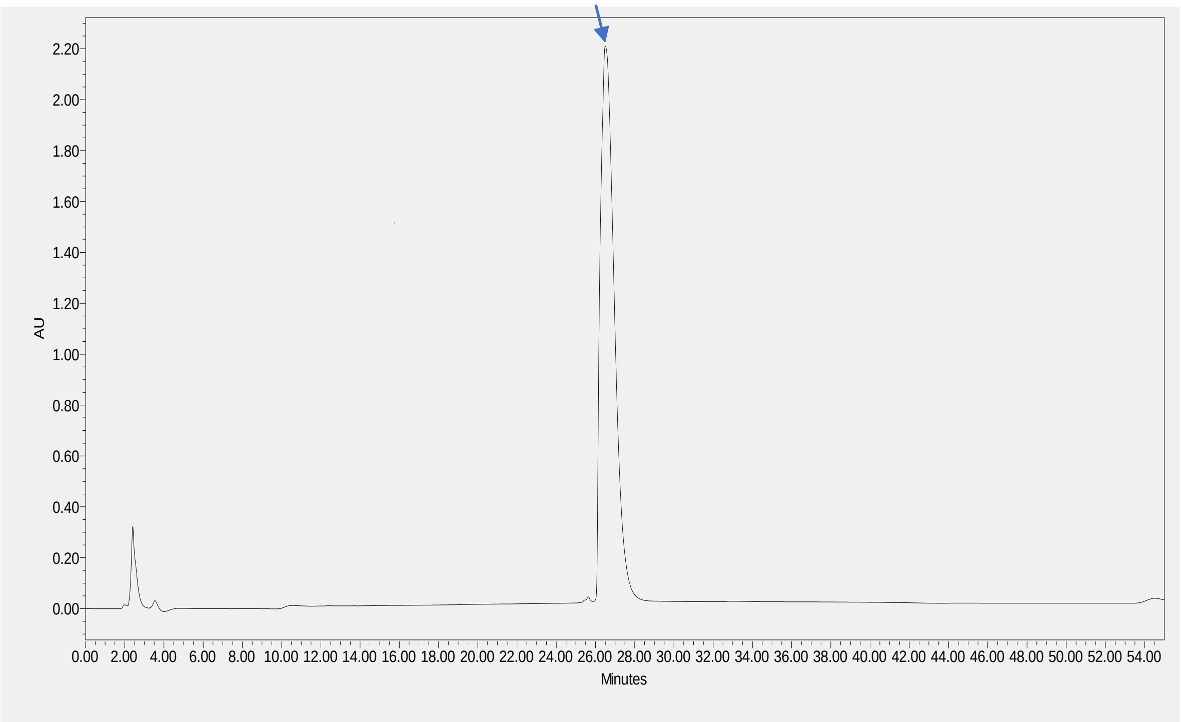

(**a**)

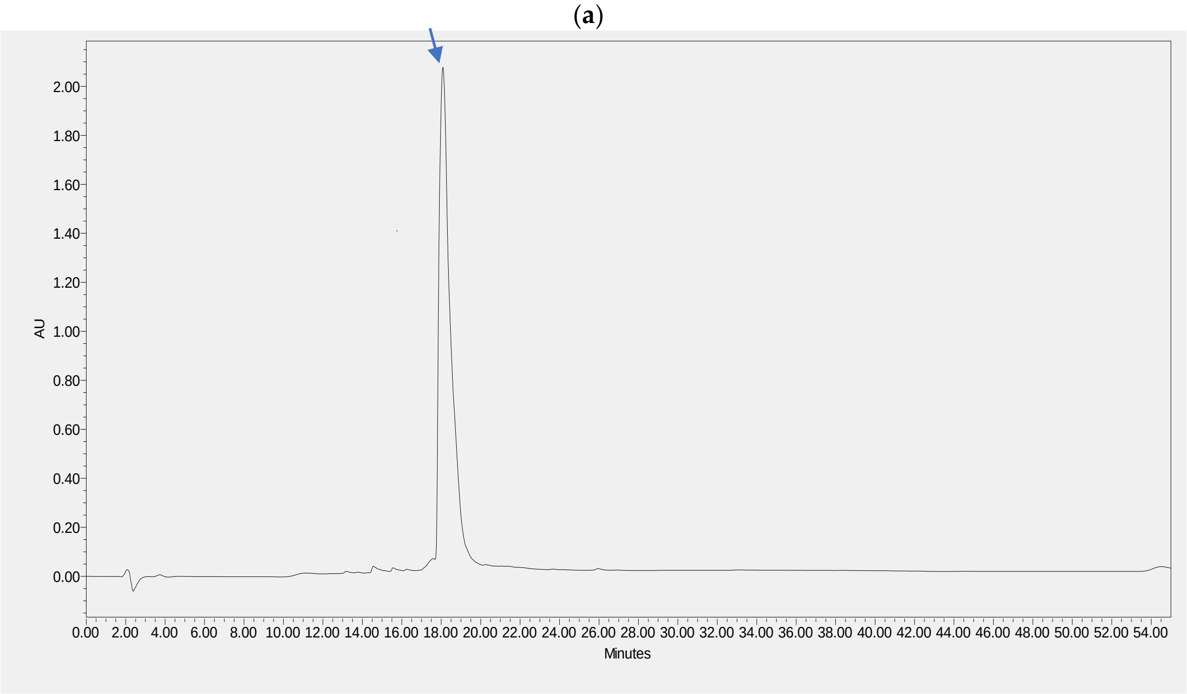

(**b**)

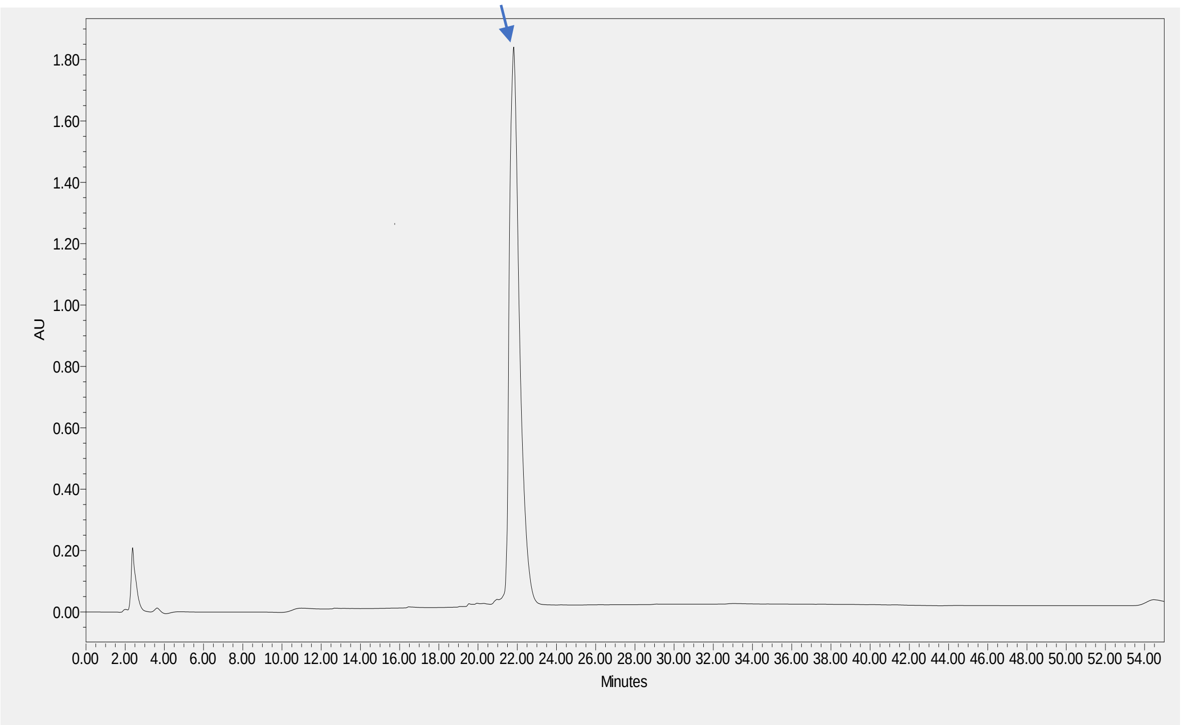
1.80
1.60
1.40
1.20
1.00
0.80
0.60
0.40
0.20
0.00
AU
0.00 2.00 4.00 6.00 8.00 10.00 12.00 14.00 16.00 18.00 20.00 22.00 24.00 26.00 28.00 30.00 32.00 34.00 36.00 38.00 40.00 42.00 44.00 46.00 48.00 50.00 52.00 54.00
Minutes

(c)

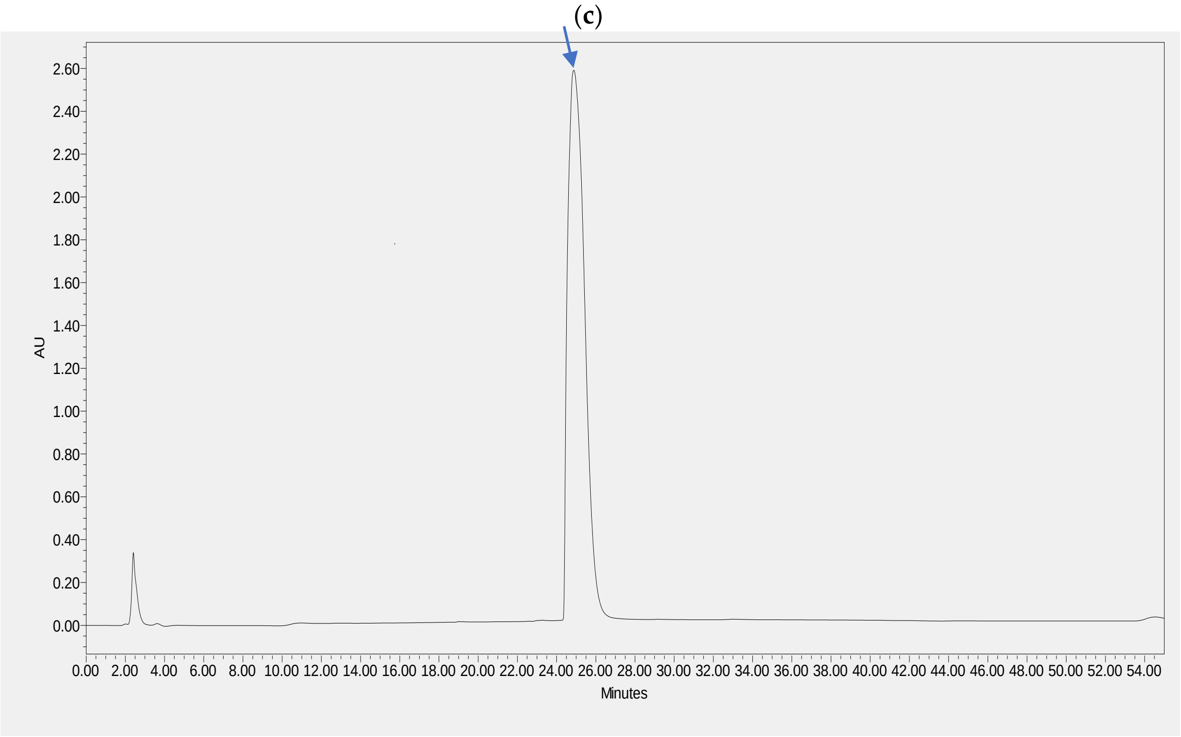
2.60
2.40
2.20
2.00
1.80
1.60
1.40
1.20
1.00
0.80
0.60
0.40
0.20
0.00
AU
0.00 2.00 4.00 6.00 8.00 10.00 12.00 14.00 16.00 18.00 20.00 22.00 24.00 26.00 28.00 30.00 32.00 34.00 36.00 38.00 40.00 42.00 44.00 46.00 48.00 50.00 52.00 54.00
Minutes

(d)

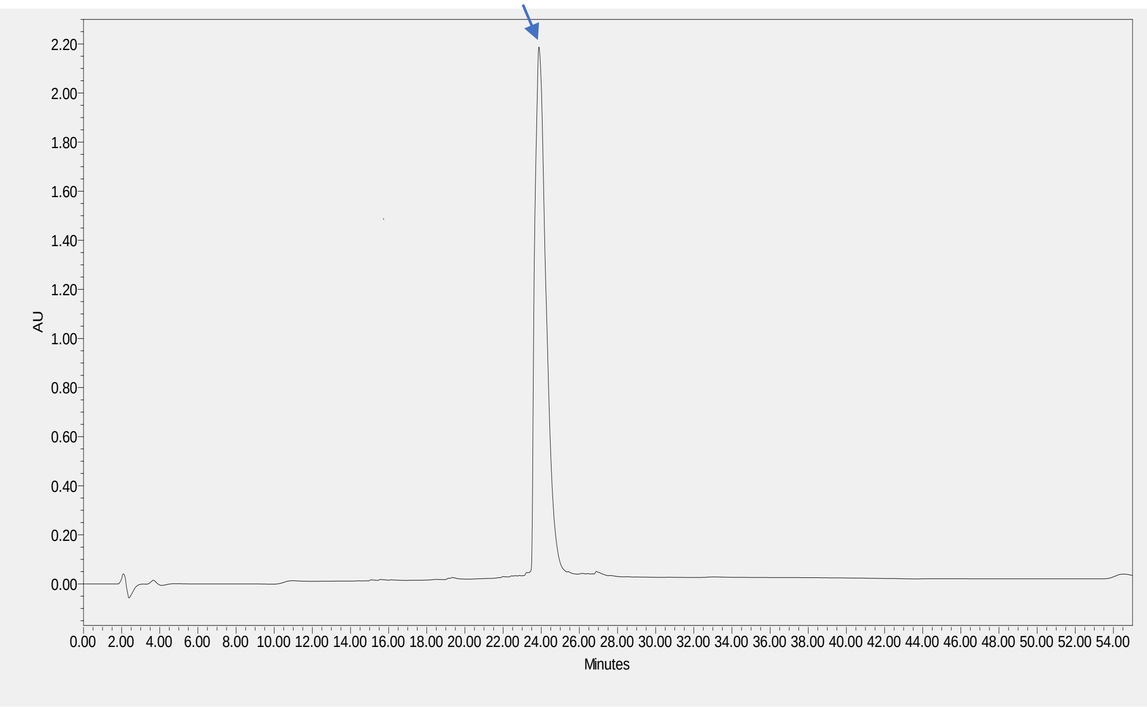

(**e**)

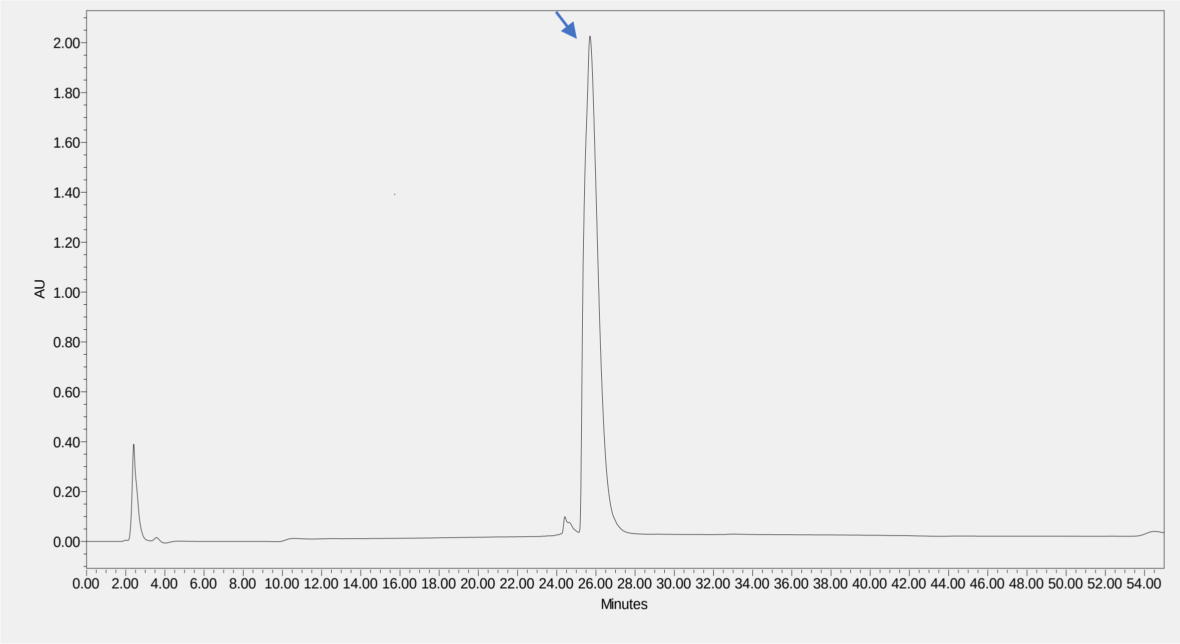

(**f**)

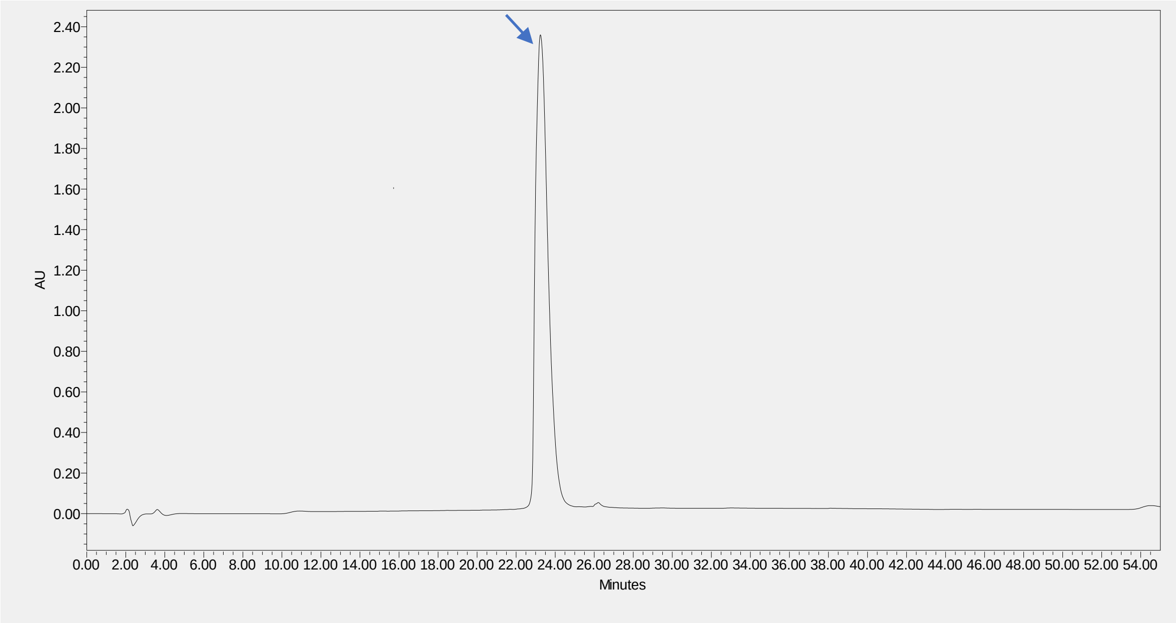

(g)

Figure S1. The RP-HPLC chromatogram of the pure peptides (QUB-1641 (a), QUB-1599 (b), QUB-1585 (c), QUB-1570 (d), QUB-1643 (e), QUB-1498 (f), and QUB-1609 (g)). The peak indicated by the arrow is the elution peak of the peptide.

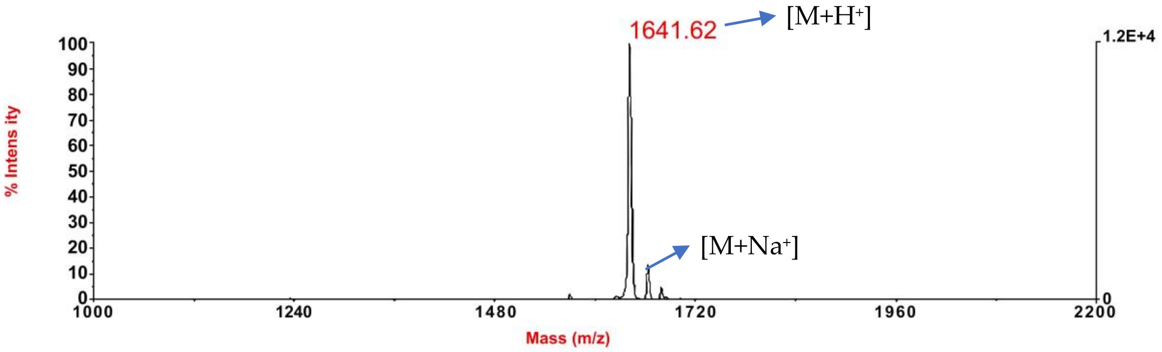

(a)

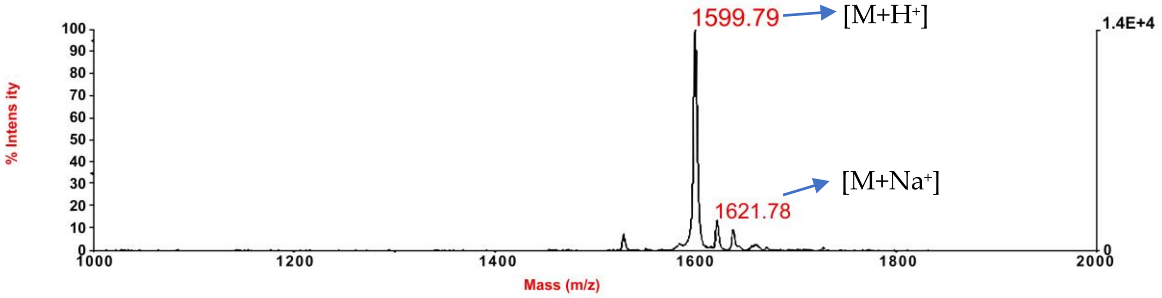

(b)

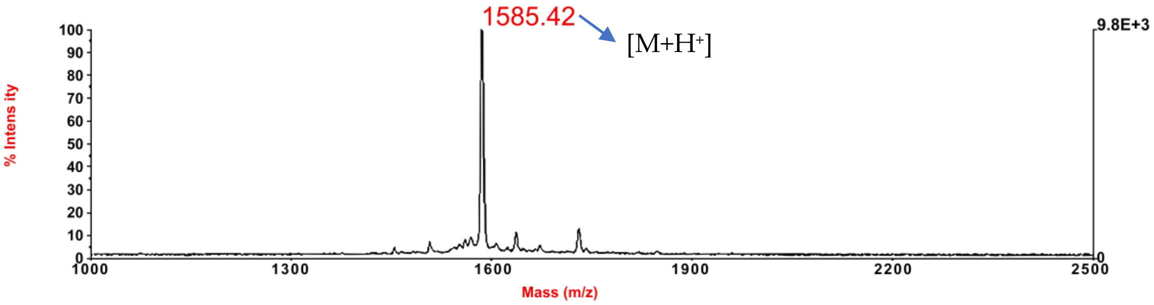
1585.42
[M+H+]
9.8E+3
% Intens ity
100
90
80
70
60
50
40
30
20
10
0
1000
1300
1600
1900
2200
2500
Mass (m/z)

(c)

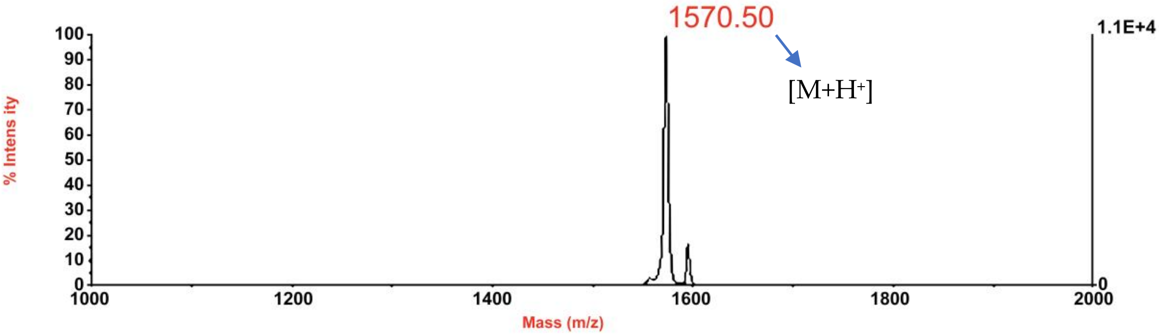
1570.50
[M+H+]
1.1E+4
% Intens ity
100
90
80
70
60
50
40
30
20
10
0
1000
1200
1400
1600
1800
2000
Mass (m/z)

(d)

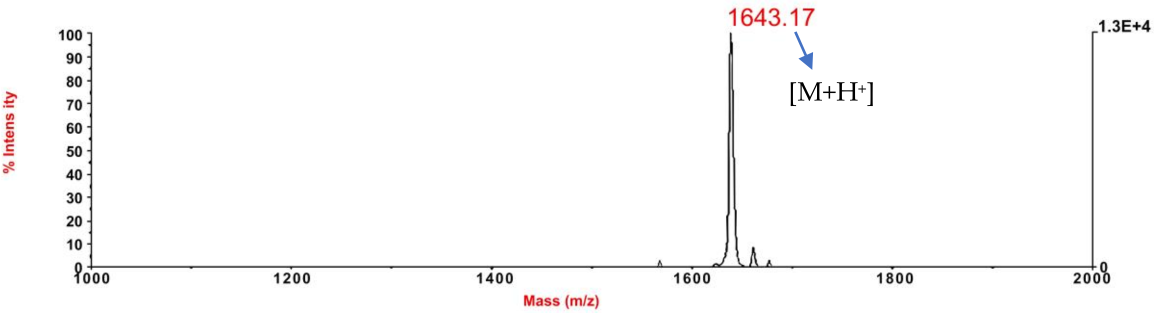
1643.17
[M+H+]
1.3E+4
% Intens ity
100
90
80
70
60
50
40
30
20
10
0
1000
1200
1400
1600
1800
2000
Mass (m/z)

(e)

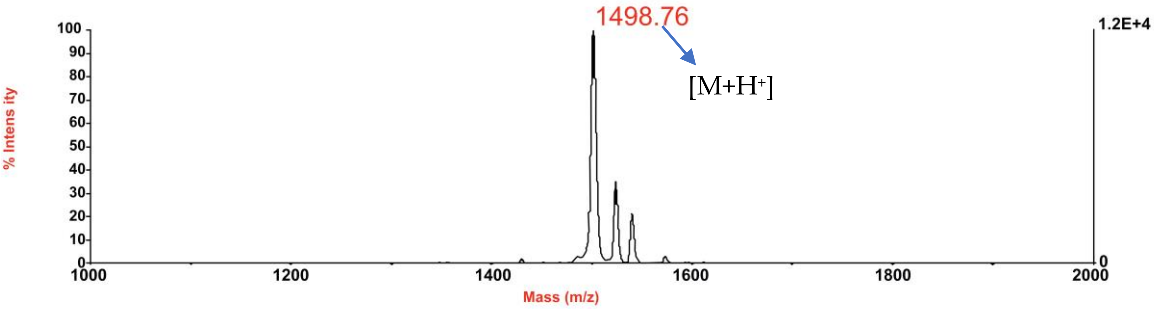
1498.76
[M+H+]
1.2E+4
% Intens ity
100
90
80
70
60
50
40
30
20
10
0
1000
1200
1400
1600
1800
2000
Mass (m/z)

(f)

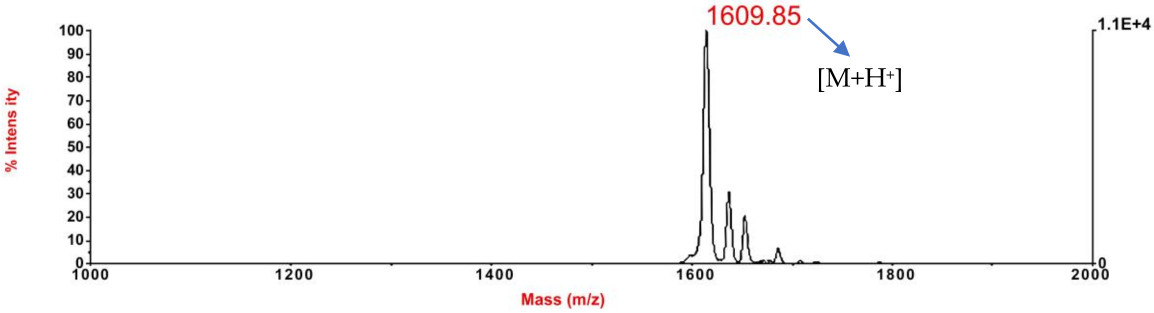
1609.85
[M+H+]
1.1E+4
% Intens ity
100
90
80
70
60
50
40
30
20
10
0
1000
1200
1400
1600
1800
2000
Mass (m/z)

(g)

Figure S2. The purified peptides' MALDI-TOF mass spectra (mass-to-charge ratios) (QUB-1641 (a), QUB-1599 (b), QUB-1585 (c), QUB-1570 (d), QUB-1643 (e), QUB-1498 (f), and QUB-1609 (g)). The peptides' [M+H$^+$] ion peaks and their sodium ion adducts were indicated by arrows.
